# Educational value of surgical telementoring

**DOI:** 10.1002/jso.26524

**Published:** 2021-07-10

**Authors:** Khayam A. Butt, Knut Magne Augestad

**Affiliations:** ^1^ Department of Gastrointestinal Surgery Nordlandssykehuset Bodø Norway; ^2^ Department of Gastrointestinal Surgery Akershus University Hospital Oslo Norway; ^3^ Department of Surgery Helgelandssykehuset, Sandnessjøen Sandnessjøen Norway

**Keywords:** mentee, mentor, surgical telementoring, telestration

## Abstract

Educating surgeons is a time‐consuming process. In addition to theoretical knowledge, the practical tasks of surgical procedures must be mastered. Translation of such knowledge from mentor to mentee may be efficiently done by surgical telementoring (ST). This is a review on surgical telementoring. Recent technological advances have made this tool in surgical education more available and applicable but future applications of ST have to be wisely guided by high‐quality trials.

## 1 INTRODUCTION

Telementoring reports for educational purposes can be traced back to the 1960s with DeBakey performing the first open‐heart surgery. Originally, there were only a few interested contributors to surgical telementoring, and the technique and hardware were not available. Advances in telecommunication technology made possible the development of low‐cost and reliable solutions for distant telementoring.^1^, ^2^ Emerging challenges, including the expanding world population and the time and resources needed for educating the surgical workforce,^3^ have foreseen surgical telementoring as a possible solution to enhance and improve surgical education. Development in the field of surgery has evolved toward minimally invasive surgery where live camera image represents the surgeon's view of the surgical field. Because ST allows the mentor to guide the mentee by live transfer of video and audio feeds of the surgical field, it has been proposed as a natural fit for surgery.^4^

The traditional Halstadian approach to surgical teaching^5^ has been challenged over the recent decades. Restrictions in allowed working hours for residents in addition to recent restrictions in international travel caused by the ongoing pandemic, has limited hands‐on sharing of surgical experience between mentors and mentees. These are some of the factors which have contributed to a paradigm shift regarding surgical teaching and education.^6^, ^7^ Video‐based surgical coaching^8^ including telementoring in surgical education holds promise in regard to improved surgical education and may efficiently allow acquisition of surgical skills.^9^

Despite numerous evaluating surveys assessing surgical telementoring over the last decades, there is still a lack of widespread application of this tool in surgical education.

## 2 DEFINITIONS

### 2.1 Surgical telementoring

According to the Society of American Gastrointestinal and Endoscopic Surgeons (SAGES), telementoring is a relationship facilitated by telecommunication technology in which an expert surgeon (mentor) provides guidance to a less experienced learner (mentee) from a remote location.^9^

### 2.2 Mentor—telementor

A mentor/telementor is an expert surgeon providing guidance during a live surgical setting to a mentee. There are defined prerequisites^10^ required for both mentor and mentee including proof of skills and experience by the involved mentor (i.e., clinical experience, teaching capabilities, LapCo experience/TT‐course). Of note is that surgical telementoring requires additional communication skills (i.e., predefined communication protocol) from both mentor and mentee due to the time lag experienced during present 4G wireless network used to transmit the video‐audio signals.

### 2.3 Mentee—telementee

A surgeon with appropriate knowledge and experience seeking individual training in a specific procedure he or she is lacking experience in. The goal is acquisition of surgical knowledge/skills and the medium used for this purpose may be telementoring.

There have also been defined prerequisites for the mentee (i.e., board eligibility and enrollment in an educational pathway in the specialty in question).^10^

### 2.4 Tools in surgical telementoring

#### 2.4.1 Live video feed

It allows continuous images from the surgical site to be shown during laparoscopic or robotic surgery. With this technological capability, surgical telementoring is considered a natural fit for the teaching process in minimal invasive surgery. The mentor is able to switch between the live video feed of the laparoscopic or robotic camera and a view provided by an external camera which might be placed at specific sites in the operating room (OR). This enables the mentor to provide a more comprehensive evaluation of the procedure, taking into account external positioning of trocars or robotic arms in addition to the positioning of the assistant surgeon and nurse.

#### 2.4.2 Live audio feed

It allows continuous instructional verbal interaction between mentor and mentee during the procedure. The verbal communication may be provided by a headset worn by the operating mentee, or the voice of the mentor may be provided through loudspeakers in the OR. The latter may give disturbance in communication but has the advantages of including the whole staff in the OR into the ongoing communication. The verbal communication should follow some rules of conduct agreed upon by the mentor and mentee. The number of verbal interactions or corrections could be regarded as a measure for the mentee's degree of needed support.

#### 2.4.3 Telestration

It allows the mentor to give instructions by freehand sketches made on a still picture taken from the continuous video‐feed. Identification of anatomical landmarks and planes of dissection might be clarified and warnings of anatomical danger zones may be given. The instructional session with telestration requires a pause in the ongoing surgery. The still picture is taken by the mentor who then may ask the mentee to stop the ongoing procedure for a telestrational session. The telestrational instructions as well as verbal feedback are shown to enhance the educational value of surgical telementoring and may reduce the telementoring session by more than 30%.^11^ The telestrational session has an inherent weakness in that it may exclusively be carried out on a still picture of the live video feed, but possible future solutions to the problem have been discussed by Budrionis et al.^12^

#### 2.4.4 Educational frameworks in surgical telementoring

It is helpful to adapt and have knowledge about conceptual frameworks in educational surgical telementoring programs. These frameworks assist the mentor and place the telementoring curriculum into a broader context and are useful in the planning and fulfilling of an effective surgical telementoring curriculum.^9^, ^13^, ^14^, ^15^, ^16^, ^17^

#### 2.4.5 Video technology and education: task‐technology fit model

The "task‐technology fit model" is defined by Maruping^16^, ^17^ as "the degree to which technology assists a group in performing its portfolio or task." The "task‐technology fit model" applies to surgical telementoring in several ways

1) High immediacy in feedback: surgical telementoring enables high immediacy in feedback to the surgical trainees as communication is live.

2) High symbol variety: video coaching offers high symbol variety in communications where live video, audio, and telestration are applied simultaneously.

3) Communication with multiple participants simultaneously.

4) High richness: richness is the ability of information to change perception within a time interval for tasks requiring social presence. High richness is seen in surgical telementoring as mentors provide immediate feedback to the mentees to check interpretation and task performance during the surgical procedure.

#### 2.4.6 The ADDIE model: planning a surgical telementoring educational program

Mentors may use the ADDIE model^13^ as a descriptive guideline in a structured surgical telementoring curriculum.

1) *Analysis* phase: defines the surgical procedure's instructional challenges and distinguishes the learning environment and the surgical trainees existing knowledge and skills.

2) Design phase: classifies the surgical learning objectives and assesses available instruments, exercises, content, analysis of the subject matter, lesson planning, and ST technology setup.

3) Development phase: mentors create storyboards and graphics to enhance learning. The mentors and mentees generate a communication protocol. The telementoring curriculum design is reviewed and revised according to feedback from the surgical trainees.

4) Implementation phase: a step‐by‐step surgical procedure for mentors and mentees is discussed and carried out according to the curriculum.

5) Evaluation phase: the evaluation phase consists of two features: formative and summative. Formative evaluation is present in each stage of the ADDIE process. After the teaching program is over, summative evaluation and 360‐degree feedback are feasible.

#### 2.4.7 The GROW model: providing structured feedback to mentees

The GROW (Goals, Reality, Options, Wrap‐up) model is used in various surgical training situations such as surgical telementoring.^15^ The GROW model proposes a way of structuring mentor sessions to facilitate a balanced discussion. The GROW model promotes a mentoring conversation through four essential stages of goal‐oriented coaching.

1) Goals: focus on specific goals that the surgical trainee wishes to accomplish during the surgical procedure.

2**)** Reality: exploration of the fundamental nature of the problem (performance review).

3) Options: express effective solutions, mainly to allow the surgical mentees to achieve their goals.

4) Wrap‐up: develop an action plan for the surgical mentee to move toward the stated goals. Examine potential technical obstacles during surgery and identify cases for improvements.

#### 2.4.8 IDEAL framework in surgical telementoring

The IDEAL framework provides five stages for development and evaluation and is recommended when introducing surgical innovations (i.e., surgical telementoring).^18^ The IDEAL framework is of importance when the faculty is planning a surgical telementoring curriculum, and consists of the following stages: Idea, Development, Exploration, Assessment, and Long term study (IDEAL):

1) *Idea:* case reports to present the idea and background describing the need for the product or innovation in question.

2) *Development*: prospective case series demonstrating feasibility and assessing safety and short‐ term effects

3) *Exploration*: prospective multicenter case series for exploration of the idea.

4) *Assessment*: randomized controlled trials are needed, including informed consent process, inclusion and exclusion criteria and description of risk factors.

5) *Long‐term study*: involves population‐based registry trials for long‐term assessment of an innovation which has already been clearly described in detail.

#### 2.4.9 The development of an ST educational curriculum

**Figure 1:**
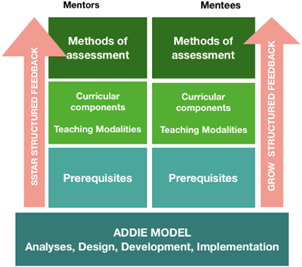
A surgical telementor curriculum. The objective of a surgical telementoring curriculum is to facilitate proficiency in a surgical technique/method [Color figure can be viewed at wileyonlinelibrary.com]

According to the SAGES ST educational task force, a structured ST curriculum consists of four main elements: (1) prerequisites for entering the program; (2) teaching modalities; (3) curricular components; and (4) assessment methods (Figure 1).^9^

1) Prerequisites: given that a mentor/mentee in a surgical telementoring program is not a pure novice, defining an entry‐level performance is relevant and should be defined in terms of knowledge, skills, and leadership. The mentor must prove excellence in the surgical procedure itself and must demonstrate high‐level knowledge and pedagogical qualifications. Mentors are by definition experts in the surgical procedure. They are experts in all aspects of surgical telementoring, including virtual communication, risk management, and leadership in a virtual environment. Similarly, the mentees should have specific predefined surgical skills and be affiliated with an accredited institution with a letter of support from their hospital.^9^

2) Teaching modalities: different teaching modalities are necessary, including online sessions, didactic lectures, interactive simulation, and telementoring simulation. For mentors/mentees in their original experience with surgical telementoring, simulated sessions are helpful. These sessions should reflect different settings such as the OR, inter‐, or intra‐hospital, or as appropriate to the planned application.^9^

3) Curricular components: principally, the goal of a surgical telementoring curriculum is not different from other surgical courses, that is, to facilitate progression toward mastery in a surgical method. However, the educational context is fundamentally different from traditional mentoring, and the curricular components should focus on the technology, including troubleshooting and communication obstacles and teamwork. Languages and terms in surgical telementoring can be different, and there is a need to develop a structured method to communicate during an ST session, including common vocabulary and communication patterns.^9^

4) Assessment methods: video‐based rehearsals may be an exceptionally efficient approach to assess a surgical telementoring sessions. Available instruments such as The Global Operative Assessment of Laparoscopic Skills (GOALS) and Operative Performance Rating Scale (OPRS) should be helpful in this setting. Other established assessment methods should also be used (360‐degree feedback, pre, and postcourse test, etc.)

#### 2.4.10 Methods of assessment in surgical telementoring

Surgical telementoring is a process in which a mentor gives constant support, enabling the mentee to adopt proficient, effectivite, and safe‐conduct during a specific surgical procedure. Several tools for assessment of the quality of the surgery have been developed.

#### 2.4.11 Assessment of technical skills

##### Video coaching

Video coaching involves the assessment of recorded videos of the surgery carried out by the mentee without intraoperative involvement of the mentor. These recorded videos may retrospectively be used for educational purposes to examine surgical performance, including technical, cognitive, and interpersonal skills. The coaches then identify individualized performance goals, evaluate current performance, and design an action plan to advance toward those goals. Video coaching might be used as an adjunct during the process of surgical telementoring. Reviews of procedures might be a particularly efficient method of assessing telementoring sessions. Video coaching might hence be regarded as an advanced part of the telementoring educational process where the mentee is first mentored by surgical telementoring with verbal and telestrational guidance during surgery and then video coaching would be utilized as a gradual part of achieving independence.

##### Scoring scales for surgical skills

The effectiveness of surgical mentoring/telementoring can be measured and qualitative feedback can be given to the surgical mentee in an educational setting. Several validated scoring scales for specific purposes have been proposed. The scoring scales are designed to give optimal evaluation for open, laparoscopic or robotic surgery. There is a strong association between video‐scored surgical performance and surgical complications. Scoring scales have been matched with quality registry, and surgical skills measured by these scales have shown significant association with the rate of complications.^19^ Application of the scoring scales for assessment of surgical skills has been shown to be reliable in the hands of experts (surgeons), nonexperts (crowdsourcing),^20^ and by automated software programs.^21^ There is an emerging trend for crowdsourcing as a quick and cheap method for assessment of technical skills. A recent systematic review and meta‐analysis of video‐based coaching in surgical education^8^ identified 13 different validated scoring scale instruments utilized in the included studies that might be feasible to use in telementoring studies. (Table 1). Of particular value in surgical telementoring are the following scoring scales:

**Table 1:** Scoring scales technical useful for assessment of surgical telementoring^8^

Name	*n*	General versus procedure specific	Components	Scale	Max points	Original reference
**OSATS GRS** Objective structured Assessment of Technical Skills: Global Rating Scale	15	G	Respect for tissue; time‐motion; instrument handling; knowledge of instruments; use of assistants; flow of operation; knowledge of procedure	5‐point Likert	35	Martin (1997); Reznick (1997)
**OSATS** + **additional metrics** Objective Structured Assessment of Technical Skills + time + number of radiology images	1	G/P	OSATS GRS + time to completion + number radiological images requested	5‐point Likert	90	Karam (2015)
**Modified OSATS** Modified objective structured assessment of technical skills	3	P	OSATS GRS + 1‐2 procedure‐specific variables	5‐point Likert	45	Reznick (1997); Karam (2015)
**BOSAT** Bariatric objective structured assessment of technical skills	1	P	Jejunojejunostomy; gastric pouch; linear stapled GJ; circular stapled GJ; hand‐sewn GJ	5‐point Likert	52	Zevin (2013)
**GERT** Generic error rating tool	1	G/P	Abdominal access, use of retractors, use of energy devices, grasping and dissection, cutting transection and stapling, clipping, suturing, suction	1 error = 1 point	Error sum	Bonrath (2013); Husslein (2015)
**Royal College of Surgeons** Wound suturing system	1	P	Task specific wound suturing checklist	Correct performance: 1 or 2 points	20	Farquharson (2013)
**GOALS** Global Operative Assessment of Laparoscopic Skills	3	G	Depth perception, bimanual dexterity, efficiency, tissue handling, autonomy	5‐point Likert	25	Vassiliou (2005)
**GOALS** + Global Operative Assessment of Laparoscopic Skills plus Vaginal Cuff Metrics	1	P	GOALS + needle handling, knot tying, vaginal mucosa incorporation	5‐point Likert	40	Rindos (2017)
**GEARS** Global Evaluative Assessment of Robotic Skills	1	G	Depth perception, bimanual dexterity, efficiency, force sensitivity, autonomy, robotic control	5‐point Likert	30	Goh (2010)
**OPRS** Operative Performance Rating System (hernia)	1	P	Ilioinguinal nerve, indirect hernia, mesh insertion, anatomy knowledge, femoral vein injury, prevention of complication, respect for tissue, time and motion, flow of operation, overall performance	5‐point Likert	50	Larson (2015)
**ASCRS scale** Right colectomy assessment scale	1	P	Exposure, vascular, mobilization, anastomosis, overall	6‐point Likert	30	Crawshaw (2016); Miskovic (2013)
**CAT/l‐CAT** Competency assessment tool (cholecystectomy/lap colorectal surgery)	1	P	Cholecystectomy: Exposure, Calots triangle dissection, Resection.	5‐point Likert	Variable	Cole (2013); Miskovic (2013)
**PQ score** Performance quotient	1	G	PQ = [Instrument handling + body position + accuracy + tightness + alignment] × percent completion	5‐point Likert and % completion	Variable	Summers (1999)

Abbreviation: GJ, gastrojejunostomy.

a. Objective Structured Assessment of Technical Skills (OSATS)

This is the most commonly used validated tool for assessment of technical skills for open surgery, utilized in 15 of the studies included in the mentioned meta‐analysis by Augestad et al.^8^ The seven domains of assessment utilized by this scoring system (1. knowledge of instruments; 2. use of assistants; 3. knowledge of specific procedures; 4. respect for tissue; 5. time and motion; 6. instrument handling; and 7. flow of operation and forward planning) are scored on a Likert Scale between 1 and 5. Domains 1–3 are not scorable due to obvious video analysis limitations. So, utilization of OSATS in video assessments can give a total score range from 4 to 20.

b. The Global Operative Assessment of Laparoscopic Skills

This is one of the most commonly used and validated assessment tools for grading technical proficiency for laparoscopic surgery. The five domains of assessment applied in this scoring system (1. depth perception; 2. bimanual dexterity; 3. efficiency; 4. tissue handling; and 5. autonomy) can be scored on a Likert scale between 1 and 5. The autonomy domain may not be scored as video analysis does not allow for evaluation of verbal guidance. Hence, the total score achieved for this scoring system can give a total score range from 5 to 25.

c. The Global Evaluative Assessment of Robotic Skills

This is the most commonly used validated assessment tool for grading technical proficiency for robotic surgery. While it measures domains such as fluidity of motion, it does not define surgical or clinical judgment. Each of the six domains utilized in this scoring system (1. depth perception; 2. bimanual dexterity; 3. efficiency; 4. force sensitivity; 5. robotic control and 6. autonomy) can be scored on a Likert scale between 1 and 5. The autonomy domain may not be scored as video analysis allow for evaluation of verbal guidance. The total achievable score by this video platform ranges from 5 to 25.

##### Assessment of nontechnical skills

Recent studies of surgical performance have shed light on nontechnical surgical skills required by the surgeon.^22^, ^23^ Surgeons are required to perform in environments where performance is dependent on ability to cooperate as a member of a team in addition to the requirements of technical surgical skills. Sometimes the surgeon becomes a natural leader of a multidisciplinary team working together to achieve the best outcome for a patient undergoing surgery.

Utilizing multisource feedback (MSF) as a method of assessment of a surgeon's abilities has been incorporated into the recertification process in several countries.^24^ This method of assessment gathers feedback from multiple individuals occupying a variety of roles in the surgeons working environment.^25^ MSF, also referred to as 360‐degree of feedback, aims to give a more comprehensive perspective on performance where peers, superiors, and subordinates give structured feedback on the surgeon's abilities.^26^ The surgeon's own opinion about his or her performance scored by the review may also be taken into account in such a global assessment. Information gathered from such surveys of an individuals' performance can be used to enhance or achieve the required code of excellence within a unit.^27^

The industry has gained interest in developing a software network for providing individual assessment of surgeons by peer‐to‐peer discussions and assessment of video‐recorded surgical procedures by expert surgeons. An example of such an industry‐driven platform is C‐SATS©.

### 2.5 Mentor assessment

Assessment of the competency of the mentor has been the focus in several studies.^28^, ^29^ MSF is one available concept for assessment of the mentor and the mentee. The Lapco‐Train‐the‐Trainers (Lapco‐TT) is a course for surgical trainers, in which delegates learn standardized teaching techniques for skills acquisition.^30^ SAGES has advised the application of a model for faculty involved in the SAGES hands‐on courses (SAGES‐HOC) as this has shown to have an impact on the educational experience of learners at SAGES‐HOC).^30^, ^31^ Recently Wyles et al.^32^ reported on a validated method for assessment of surgical teaching. The authors developed a structured feedback tool that assesses training quality and provides feedback to surgical trainers. Twenty‐nine surgical trainers, ten trainees, and four educationalists were incorporated into semistructured interviews. Through the Delphi process, essential items pertaining to desirable trainer characteristics were determined. An assessment tool called Structured Training Trainer Assessment Report (STTAR) was tested for feasibility, acceptability, and educational impact. A web‐based miniversion of the STTAR was subdivided into four assessment groups: (1) “teaching structure”; (2) “teaching behavior’’; (3) ‘‘mentor attributes’’; and (4) ‘‘role modeling.’’ The authors have conclusively presented an assessment tool evaluating mentor quality and STTAR has been successfully implemented into the English National Training Program for laparoscopic colorectal surgery. The role of STTAR in a structured surgical telementoring program needs further exploration and validation.^9^

Surgical telementoring–how to avoid pitfalls

There are several pitfalls associated with surgical telementoring, that faculty should avoid.

1) *Data security:* transfer and handling of sensitive patient information during a surgical telementoring session should be in accordance with HIPAA (Health Insurance and Accountability act).

2) Bandwidth and latency: availability and quality of network connection should fulfill requirements of bandwidth and latency. The minimum bandwidth for telementoring is advised to be 40 Mb/s. Telementoring may be achieved with lower bandwidth but high‐quality audio and video might lag and be choppy.^33^

3) *Image quality:* ideal resolution for image quality in sophisticated telementoring applications is recommended to be 1080 progressive scan (1080 p) at 30 frames per second.

4) *Maximum latency:* for live telementoring with the possibility of annotation (telestration), SAGES technology working group has recommended a total latency of less than 450 ms.^33^

5) *Level of expertise:* a surgical telementoring initiative should be initiated by clarification of goals of the initiative and is not recommended for novice mentees.^9^

6) *Predefined mentor:* mentee contract: Both the mentor and mentee are recommended to agree upon a mentor‐mentee contract^9^ before beginning a surgical telementoring initiative. There should be an appreciation of feedbacks and the acceptance of the session being an arena for learning and teaching.

7) *Onsite versus telementoring training:* gradual approach to a surgical telementoring session is preferable. Starting off with on‐site mentoring and proceeding with virtual sessions is recommended.

8) *Develop a predefined communication protocol:* surgical telementoring systems will have certain communication obstacles. Some degree of voice delay in the verbal communication between the mentor and mentee may create frustration. Establishing rules of communication may minimize these risks.

9) *Start with the easy cases:* start off with preoperative selection of patients by excluding patients in which intraoperative challenges in addition to the planned procedure may be anticipated (i.e., required adhesiolysis because of earlier surgery and difficulties because of high Body Mass Index).

10) *Avoid logistic obstacles:* logistic obstacles may be minimized by applying a preoperative surgical telementoring technology checklist.

### 2.6 Examples of ongoing telementoring initiatives

Resent research in the field of surgical telementoring has highlighted the need of randomized controlled trials exploring educational outcomes.^9^ A structured educational curriculum utilizing surgical telementoring is required in these trials. The authors present two ongoing trials that assess a predefined and stepwise surgical telementoring curriculum (Figure 1).

### 2.7 Robotic ventral mesh rectopexy (RVMR) initiative

This initiative was born because of the need of surgical experience in RVMR in a medium‐sized hospital, in Norway located in the north of the country where the patient population is relatively scarce. Developing experience in the technique of RVMR in such a setting was challenging as the learning curve might be prolonged because of reduced patient access. In addition, access to an on‐site mentor was difficult as no existing surgeon at the hospital had experience with laparoscopic or RVMR, and the hospital location was remote.

**Figure 2:**
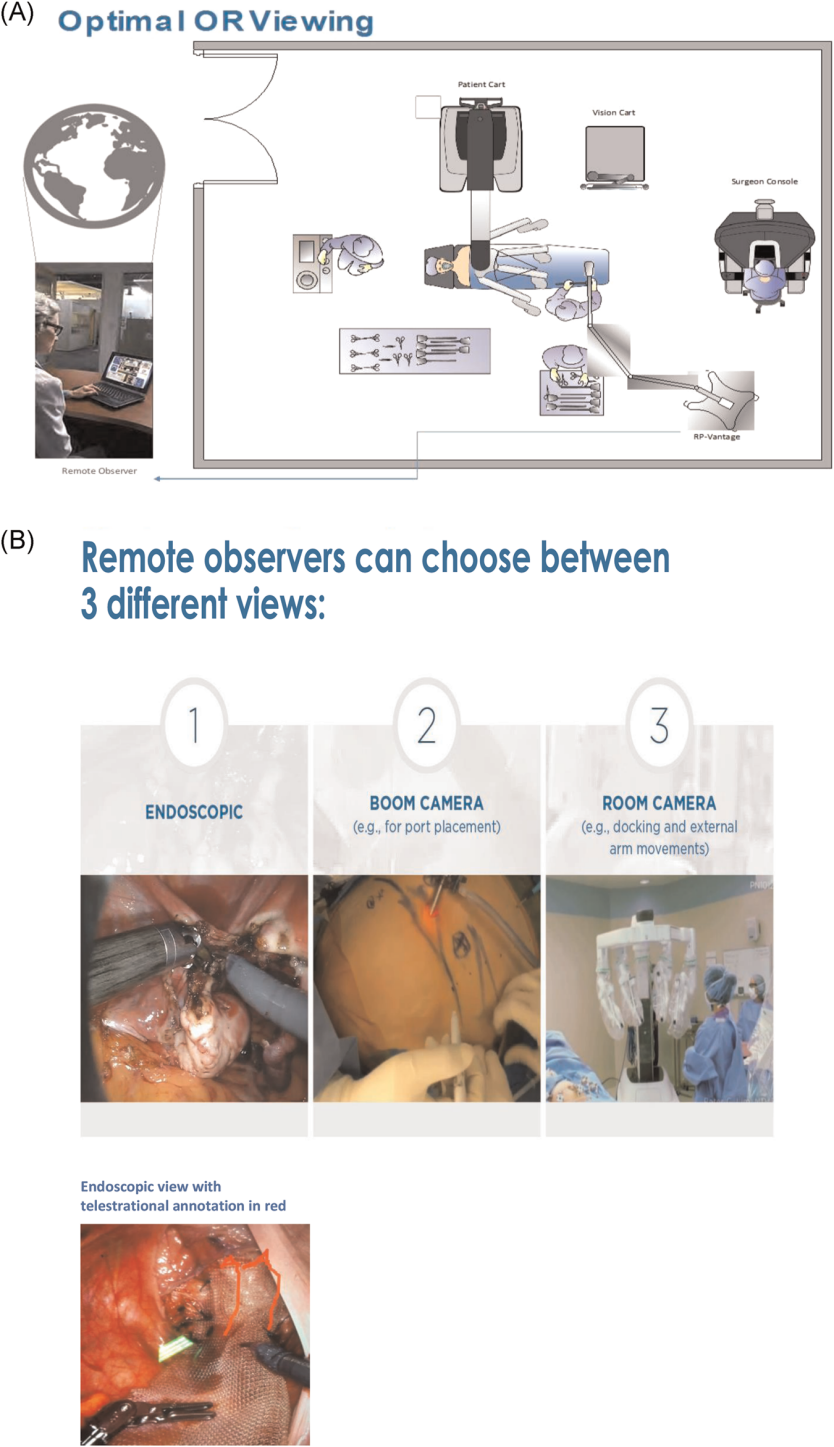
(A) The InTouch RP, Viewpoint System with Surface Pro 4 tablet. Enabling a real‐time broadcast of the procedure done by the mentee under supervision of a remote mentor.(B) Observational views for the mentor during robotic surgical telementoring [Color figure can be viewed at wileyonlinelibrary.com]

The solution for the author, being a colorectal surgeon with sufficient experience in laparoscopic and robotic colorectal cancer surgery, was to seek experienced mastery from another institution. After visiting the founding father of laparoscopic VMR, prof André D'Hoore in Leuven, Belgium, an initiative for a telementor‐guided learning process in this procedure was organized. Dr N. Thomassen, having ample experience in RVMR in addition to being a practicing mentor in DaVinci surgery, was selected as the telementor. Dr K.M. Augestad, with his vast background in the field of telementoring gave his support in conceptualizing the study. Additional experienced robotic surgeons functioned as an expert panel assessing the video recordings of the telementored surgeries. The mentee, Dr Khayam Butt, was assessed in the telementoring guided procedures of RVMR. Patient inclusion to the standardized procedure of ventral mesh rectopexy was initiated by on‐site mentoring of two procedures carried out at the mentee's institution with the mentor approving the mentee and the institution for further telementoring guidance. Preliminary results support the notion of surgical telementoring being a quality‐enhancing tool in the process of surgical education involving safe and efficient acquisition of skills in a new surgical procedure. Figure 2A shows the setup and Figure 2B shows the setup and telestrational annotations during the telementored RVMR‐initiative.

### 2.8 Experience of the Medprescence© laparoscopy initiative

Laparoscopic cholecystectomy (LC) is one of the most frequently performed surgeries in general surgical wards. Being amongst the first complicated operations carried out by general surgical residents, it is a potentially complicated procedure with complications which might have profound effect on patient outcome. Hence, it is a procedure in which surgical residents need ample time and efficient volume to achieve independence. After obtaining substantial level of skill in laparoscopic surgery, the impression in daily surgical practice is that registrars often need to call in a more experienced consultant for advice and technical support. In a busy practice, it may be difficult to obtain such support immediately, and both the operation time and the quality of surgery may be affected.

We aim to construct an educational program for a selected group of residents performing laparoscopic cholecystectomy under the guidance of a telementor with pre‐, per‐, and postoperative guidance. We seek to assess efficiency and safety of telementoring as a tool for skill development compared to traditional onsite mentoring. We will be conducting a three‐center noninferiority trial assessing two parallel groups. The intervention group is registrars receiving telementored intraoperative supervision compared to the control group being registrars receiving conventional in‐person mentoring in the OR. The randomization process will be performed as block randomization with 1:1 allocation.

Before performing such a compound trial, we assessed the safety and applicability of telementoring technology by Medprescence© as a tool for skill development among a group of surgical registrars performing laparoscopic cholecystectomy in a pilot study.

The objective of the pilot study is to obtaining a “proof of concept” before conducting the randomized controlled trial, enabling us to anticipate pitfalls.

The skill‐enhancing benefits of a telementor‐guided procedure involving the above‐mentioned telementoring technology will be evaluated in the pilot study. There will be an emphasis on benefits and drawbacks with the technology utilized for this purpose. We will secondarily evaluate the satisfaction of both the mentor and mentee according to their experience with the telementor‐guided procedures.

The pilot will be conducted by letting two mentees (general surgical registrars) perform five consecutive telementor‐guided LCs involving two telementors. Primary outcomes will be GOALS scores of the mentees to assess skill development and secondary outcomes will be satisfaction scores of the mentees and the mentors

The pilot study has been approved by the national ethical committee and is due to initiate patient inclusion by summer 2021.

**Figure 3:**
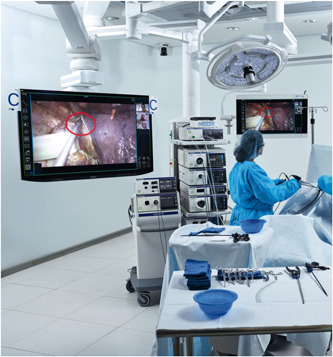
Surgical telementoring in laparoscopic cholecystectomies. Used by permission of Medprescence© [Color figure can be viewed at wileyonlinelibrary.com]

Figure 3 shows the setup in the planned laparoscopic initiative.

## 3 CONCLUSION

Telecommunication technology has made substantial improvements and the application of this technology has found new grounds into the everyday life of ordinary people. Smartphones are being used in patient follow up after cancer surgery,^34^ and there is a developing research area concerning preoperative surgical optimization of patients by use of digital phenotyping.^35^ Telecommunication technology has made its entry into the OR by telementoring applications provided by the telecommunication industry. We have witnessed an expansion from basic video records of intraoperative surgical activity for educational purposes to surgeons' sophisticated guidance in complicated surgical procedures. The emerging application of 5G new radio technology can deliver a theoretical download speed of 20 Gb/s compared to less than 2 Gb/s for current 4G LTE technology. In the future, there will be a more and better information‐transfer in near real‐time. Given the increasing availability of surgical robots in combination with 5G technology, telesurgery is no longer a futuristic thought but a current and practical application.^36^

Telementoring applications have been described as technology driven^28^ and the incredible speed of technology evolution is allowing expanded utilization. In areas such as northern Norway, there are long distances between hospitals providing certain healthcare services. Consequently, there is an increasing interest for exploring the utilization of surgical telementoring in new scenarios such as acute surgical settings, aiming to create cooperation between surgeons who are distantly located and possessing varying degrees of experience. There is limited knowledge of the implications of such utilization of surgical telementoring. The question of liability of the on‐site surgeon (mentee) and mentor may be problematic.^37^ Hospital bureaucrats and leadership may have diverging views on this topic compared to healthcare providers, such as surgeons, who may be put in complicated decision‐making situations which might have dire consequences for patient outcome. There is an obvious need for good quality research being the main guide for the expanding utilization of surgical telementoring technology in clinical practice. Studies assessing safety and feasibility of surgical telementoring in new areas of implementation should be in accordance with the IDEAL framework.^18^ Adherence to the mentioned principles is crucial to further development of this novel educational tool.

## CONFLICT OF INTERESTS

Khayam Butt has received equipment and software from Intuitive Surgical© and MedPrescence© in relation to the presented trials. The other author declares that there is no conflict of interest.

## SYNOPSIS

Surgical telementoring is a promising tool in surgical educational setting. There are however challenges which the telementor, telementee, and the faculty has to be familiar with. Standardization of communication and procedure is one of the key points in addition to a common understanding of the aim of the telementoring initiative. The technological advances over recent years and the recent pandemic have made this tool for surgical education more appliable and the demand is obvious. The future applications of surgical telementoring are vast and good quality research is required for guidance.

## Data Availability

The data which is stated in this article are available from the corresponding author upon reasonable request.
